# Excision of the Inferior Maxillary Bone for Caries

**Published:** 1853-07

**Authors:** W. G. Bullock

**Affiliations:** Savannah, Geo.


### ARTICLE XIV.

*Excision of the Inferior Maxillary Bone for Caries.* By W. G. Bullock, M. D., of Savannah, Geo.

Towards the end of July, 1852, John Turner, an Irishman, entered the Savannah Hospital, and applied to me for relief under the following circumstances.

The right side of the lower jaw, from the symphysis to the neighborbood of the articulation, was diseased, and so extensively enlarged from infiltration of the soft parts as to produce great swelling and deformity of the face, and to impede much the movements of mastication. It also rendered him an object of disgust to himself and others, in consequence of the sanious and excessively fetid discharge which flowed abundantly from several fistulous orifices opening internally in the mouth, and externally on the cheek. A probe introduced into these fistulæ, discovered the bone not only denuded, but so perforated and broken up, that the instrument could readily be passed into the mouth in various directions.

He was rather obtuse in intellect, and therefore I could not obtain any very satisfactory information from him of the nature or history of the disease, except that he stated, to use his own language, "he got it hurted some year or two ago."

From the nature of the case, it was evident that entire removal of the diseased portion of bone was the only remedy, and several of my professional friends who saw the case with me, were decided in their opinion as to the necessity of such an operation, other modes of treatment offering no hope or prospect of cure in our opinion.

An operation being determined upon, with the assistance of Drs. Kollock, Howard and Warner, after administering a mixture of chloroform and ether, and bringing the patient completely under the anæsthetic influence of those agents, I proceeded to perform the operation in the following manner:

My first incision was made a line or two to the left of the middle of the lower lip, by transfixing it with the knife directed obliquely upwards and backwards, then reversing the cutting edge of the knife, and continuing the incision down to the lower margin of the jaw. From the termination of that incision I boldly drew the scalpel along the entire inferior margin of the bone as well as it could be defined in the swollen state of the parts, to the angle of the jaw, turning up behind that point, and extending another incision at right angles or so with that, to a point nearly opposite the articulation. The formidable flap made by these incisions was next dissected up, and reflected upon the upper part of the face, so as to expose fully the diseased portion of bone. The jaw was then sawed through with the chain saw immediately to the left side of the symphysis. Seizing the end thus sawed through, the section of the bone was then carefully separated from its internal soft attachments, by drawing the scalpel along its internal surface with the edge close upon the bone. Considerable difficulty was experienced in detaching the soft parts about the angle of the jaw, and the advantage to be derived in using it as a lever was lost by its giving way and separating from the ramus and processes above. These had to be seized singly with a forceps, and separated by drawing them out and relieving them from their attachments above with a knife passed carefully under the arch of the malar bone. After excising portions of the diseased soft parts connected with the bone, tying the vessels cut, of which there was but one of importance, viz. the facial, and suppressing the hemorrhage, otherwise inconsiderable in this case, the flap was brought down, and the edges of the wound were accurately adjusted by means of a few hair-lip pins and adhesive plaster, and thus kept in complete apposition. Water-dressing was applied, and such other treatment adopted as circumstances required to facilitate union and healing of the wound.

He is now, at the time of writing this description, a month after the operation, going about, cheerful, and almost entirely well—union so completely established as to leave little external deformity, and exhibiting but slight traces of so extensive an operation.

